# Mental Health Status of Healthcare Workers During the Coronavirus Disease 2019 Pandemic: A Survey of Hospitals in Shiraz, Iran

**DOI:** 10.31661/gmj.v12i.2512

**Published:** 2023-02-17

**Authors:** Arash Mani, Mani Kharazi, Mohammad Reza Yousefi, Ali Akbary, Morteza Banakar, Hossein Molavi Vardanjani, Leila Zarei, Mohammad Khabaz Shirazi, Seyed-Taghi Heydari, Kamran Bagheri-Lankarani

**Affiliations:** ^1^ Research Center for Psychiatry and Behavioral Sciences, Shiraz University of Medical Sciences, Shiraz, Iran; ^2^ Health Policy Research Center, Institute of Health, Shiraz University of Medical Sciences, Shiraz, Iran; ^3^ Department of Psychiatry, Faculty of Medicine, Mashhad University of Medical Sciences, Mashhad, Iran

**Keywords:** COVID-19, Health Care Workers, Mental Health, Anxiety, Depression, Insomnia, Post-Traumatic Stress Disorder

## Abstract

Background: Healthcare workers (HCWs) directly or indirectly involved in the coronavirus disease 2019 (COVID-19) treatment process may experience severe mental consequences of the pandemic. Hence, this study aimed to evaluate the mental health status of HCWs in hospitals affiliated with Shiraz University of Medical Sciences, Iran. Materials and Methods: This cross-sectional study was performed on 503 HCWs from five hospitals in Shiraz, including one COVID-19 front-line hospital, two COVID-19 second-line hospitals, and two without COVID-19 wards. Then, to assess the levels of anxiety, depression, insomnia, and post-traumatic stress disorder (PTSD) among HCWs, the Persian versions of the Hospital Anxiety and Depression Scale (HADS), Insomnia Severity Index (ISI), and Global Psychotrauma Screen (GPS) questionnaires were placed, respectively. Results: The mean age of participants was 33.94±8.26 years, and 252 (50.1%) were females. Anxiety, depression, insomnia, and moderate to high levels of PTSD were observed in 40.4%, 37.8%, 24.5%, and 71% of participants, respectively. A history of mental disorders was associated with all four outcomes (P0.05). Females gender and living with elderly and/or children were correlated with anxiety and PTSD (P0.05). Working at COVID-19 front- and second-line hospitals were similarly linked to higher insomnia and PTSD levels (P0.05). Also, working in COVID-19 wards or non-clinical settings was associated with anxiety and depression (P0.05). Conclusion: Most of the HCWs in this study may experience mental difficulties. Some factors may increase their risk of experiencing these difficulties. Hence, in the crisis era, mental health monitoring and identification of groups with predisposing factors are required to provide appropriate care as quickly as feasible.

## Introduction

In December 2019, a highly infectious acute respiratory syndrome caused by a novel coronavirus (SARS-CoV-2) was identified in Wuhan, China. On March 11, 2020, the World Health Organization declared coronavirus disease 2019 (COVID-19) a pandemic [1].

Nowadays, the pandemic is undoubtedly one of the most stressful events, which poses a significant challenge to the social, economic, and, above all, the psychological resources of the populations [2][3][4].

Due to their direct contact with the disease, healthcare workers (HCWs) are concerned about disease transmission to their families. Lack of personal protective equipment in healthcare departments and long working hours make them especially vulnerable to emotional distress during the current COVID-19 pandemic [5].

Unfortunately, there have been reports of suicide among HCWs due to the psychological pressures of the pandemic [6]. The mental well-being of HCWs can significantly impact their ability to provide standard services for patients and the efficiency of the healthcare system, especially in situations such as the current pandemic [7].

In a study in Italy, among HCWs who were directly or indirectly engaged in providing care to COVID-19 patients, depression was reported in 24.73%, anxiety in 19.8%, insomnia in 8.27%, and post-traumatic stress disorder (PTSD) in 49.38% [8].

Also, younger age, female gender, working in front-line hospitals, and having a colleague deceased or hospitalized due to COVID-19 were associated with more mental health symptoms [8].

However, Lai et al. [9] showed that the prevalence of depression, anxiety, insomnia, and PTSD was observed in 50.4%, 44.6%, 34%, and 71.5% of HCWs, respectively. Also, female gender, and working in front-line hospitals were associated with increased mental health disorders [9].

In addition, a systematic review by Muller et al. showed that the impact of the COVID-19 pandemic on the mental health of HCWs was not limited to those working in the front-line hospital, and HCWs in various fields, positions, and exposure risks were presented with mental disorders [10].

Another study in Italy showed that prolonged presence in front-line COVID-19 hospitals was associated with increased mental health symptoms [11]. Also, symptoms of depression, anxiety, and insomnia diminished among HCWs from the pandemic onset over time [11].

Iran is one of the countries most affected by the COVID-19 pandemic, and its first wave was reported in late March 2020 [12]. Since then, five other waves have been officially reported, and the sixth and final wave-during which this study was performed-was caused by the relatively more contagious variant of Omicron (B.1.1.529) [13].

From the beginning of the pandemic in Iran, the Iranian health policymakers decided to dedicate some hospitals permanently and exclusively to patients, which caused the HCWs of these hospitals constantly exposed to COVID-19 (the group with consistent exposure).

Despite this decision, during the peaks of the disease, authorities had to transform some wards of other hospitals into COVID-19 units to increase the hospitalization capacity of patients.

Naturally, as a wave subsided, these wards returned to their former state.

As a result, HCWs of such units were only exposed to COVID-19 patients at certain times (the group with episodic exposure).

Although some other studies in Iran have tried to evaluate the impact of the COVID-19 pandemic on the mental health of the HCWs, to the best of our knowledge, no other study has tried to evaluate the mental health status of HCWs in Shiraz hospitals.

Furthermore, as far as we are aware, no other study in Iran has compared front- and second-line hospitals with and without COVID-19 wards.

In addition, few studies focused on assessing the mental health of the hospital staff who work in non-clinical sectors (such as administrative, security, and maintenance staff). Therefore, this study aimed to evaluate the impact of being directly or indirectly engaged in treating patients on the mental health status of HCWs in Shiraz hospitals.

## Materials and Methods

Study Design

In this cross-sectional study, five hospitals in Shiraz city, including Ali-Asghar, Namazi, Faghihi, Hafez, and Dastgheib hospitals, all affiliated with Shiraz University of Medical Sciences, were selected.Ali-Asghar hospital is the front-line COVID-19 hospital in Shiraz, and its staff has been continuously exposed to COVID-19 patients during the past two years.Due to the insufficient capacity of Ali-Asghar hospital at the peak of COVID-19 waves, it was decided to temporarily allocate some wards of Namazi and Faghihi hospitals (as the second-line COVID-19 hospitals) for COVID-19 patients.Consequently, during some episodes over the past two years, some hospital staff were directly exposed to COVID-19 patients. Also, Dastgheib and Hafez hospitals have no COVID-19 wards.

Participants

Based on the Rayani et al. study [14], 40% of healthcare experienced moderate and high levels of anxiety; the sample size was calculated as 276 (α=0.05, β=0.8, and d=0.06). For more accuracy, the sample size was considered as 503.Random stratified sampling was used to recruit 142 participants from Namazi, 140 from Faghihi, 120 from Ali-Asghar, 51 from Hafez, and 50 from Dastgheib hospitals, from February 1 to February 20, 2022, in the middle of the sixth wave of COVID-19 in Iran caused by the Omicron variant.Also, HCWs younger than 18 or older than 65 years were excluded from the study. Participants answered a mental health assessment questionnaire in their workplace hospital with a trained interviewer.

Data Collection

The baseline characteristics of participants included gender, age, educational level, occupation, marital status, living with children (under ten years old), living with the elderly (over 60 years old), experiencing the death of relatives or colleagues from COVID-19, history of psychiatric disorders, workplace hospital, and workplace ward of HCWs.Then, to assess the levels of anxiety, depression, insomnia, and PTSD among HCWs, the Hospital Anxiety and Depression Scale (HADS) [15], Insomnia Severity Index (ISI) [16], and Global Psychotrauma Screen (GPS) questionnaires were applied, respectively.Kaviani et al. [17] proved the validity and reliability of the 14-item HADS questionnaire and determined specific cut-off points, considering the cultural differences of the Iranian population.Also, the reliability and validity of the Persian ISI questionnaire were proved by Yazdi et al. [18].Also, the reliability and validity of the Persian version of the 22-item GPS questionnaire were proved by Haghi et al. [19].The GPS questionnaire scores were interpreted through a specific statistical method (latent class analysis) to categorize participants with the same pattern of responses to three groups with low, moderate, and high levels of PTSD.

Ethical Considerations

This study was approved by the Research Ethics Committee of Shiraz University of Medical Sciences (approval code: IR.sums.med.rec.1400.572).At the beginning of the interview, after explaining the research objectives, written informed consent was obtained from all participants. The questionnaires were completed anonymously, preserving all the principles of confidentiality.

Statistical Analysis

A data-driven approach was used to categorize the level of PTSD assessed by the GPS questionnaire.A latent class analysis (LCA) was employed to categorize participants with the same pattern of responses to questionnaires. The LCA assigns an individual to a class by examining the pattern of categorical data using probabilistic methods.Briefly, in the first step, several non-inclusive classes with homogeneous participants were defined. Then, LCA was done with the number of classes from 2 to 10.The lower Bayesian information criterion (BIC), Akaike’s information criterion (AIC),and clinical interpretability determined the number of extracted GPS classes. Therefore, three classes for GPS with the lowest level of BIC to ease the interpretation were selected. Latent GOLD (version 5.0.0) was used to perform LCA.Also, IBM SPSS Statistics for Windows, version 21 (IBM Corp., Armonk, NY., USA) was used to perform all statistical analyses. Quantitative and qualitative variables were described by mean±standard deviation (SD) and frequency (percent), respectively.Univariate and multiple logistic regressions were performed to compute the odds ratio (OR) and the corresponding 95% confidence interval (CI) for demographic features, COVID-19 infection death, psychiatric disorder, hospital features with anxiety, depression, and insomnia. A P-value less than 0.05 was considered as statistically significant.

## Results

Of the 503 participants, 252 (50.1%) were female, and the mean age was 33.94±8.26 years (ranged 20-60 years). The frequency of anxiety, depression, and insomnia was 40.4%, 37.78%, and 24.5 %, respectively. Additionally, based on the LCA method with three classes, 146 (29%), 249 (49.5%), and 108 (21.5%) participants had low, moderate, and high levels of PTSD, respectively. The class with a low level of PTSD had a mean score of 2.52±1.4 and a median of 2 (ranged zero to 6). The class with a moderate level of PTSD had a mean score of 8.11±1.95 and a median of 8 (range 4 to 11). The mean score of the class with a high level of PTSD was 14.10±2.15, with a median of 14 (range 11 to 21). The reliability of HADS, ISI, and GPS questionnaires were 0.886, 0.919, and 0.826, respectively.

Mental Health Status

1. Anxiety

Based on univariate logistic regression, gender (female: OR=1.72), occupational (non-clinical staff vs. physician: OR=1.82), living with the elderly (OR=1.57), death from COVID-19 in the relatives (OR=2.81), positive history of psychiatric disorders (OR=2.66), workplace hospital (Ali-Asghar vs. Hafez: OR=2.73), and workplace ward (working in COVID-19 wards vs. non-COVID-19 wards: OR=4.07; working only in non-clinical sectors vs. working in non-COVID-19 wards: OR=3.04) were significantly associated with anxiety (Table-1). However, as mentioned in Table-1, in multiple logistic regression, gender (female: OR=2.49), living with the elderly (OR=1.88), positive history of psychiatric disorders (OR=3.6), and workplace ward were significantly associated with anxiety.

2. Depression

Based on univariate logistic regression, age (OR=2.12), occupation (non-clinical staff vs. physician: OR=1.85), living with children) (OR=1.53), positive history of psychiatric disorders (OR=1.73), workplace hospital (Ali-Asghar vs. Hafez: OR=3.14), and workplace ward were significantly associated with depression (Table-2). Also, in multiple logistic regression, level of education, positive history of psychiatric disorders, and workplace ward were significantly associated with depression (Table-2).

3. Insomnia

As shown in Table-3, univariate logistic regression revealed that death from COVID-19 in the relatives, positive history of psychiatric disorders, workplace hospital, and workplace ward) were significantly associated with insomnia.In addition, multiple logistic regression indicated that level of education, positive history of psychiatric disorders, and workplace hospital were significantly associated with insomnia (Table-3).

4. PTSD

Based on multinomial logistic regression, living with children (OR=2.27), living with the elderly (OR=2.09), positive history of psychiatric disorders (OR=3.13), and workplace ward were significantly associated with a moderate level of PTSD (Table-4). However, the high level of PTSD was significantly associated with gender (female: OR=3.8), living with children (OR=2.92), working night shifts (OR=5.45), positive history of psychiatric disorders (OR=5.45), and workplace hospital (Table-4).

**Table:** Table **1.** Association Between Socio-Demographic Features, Death from COVID-19, Psychiatric Disorders, and Workplace Features with Anxiety among HCWs

**Variables**		**Anxiety**		**Unadjusted OR** **(95% CI)**	**P** **value**	**Adjusted OR** **(95% CI)**	**P** **value**
		**No or mild n(%) **	**Moderate to High n(%) **				
**Gender**	Female	134 (53.17)	118 (46.83)	1.72 (1.2-2.47)	0.003	2.49 (1.45-4.28)	0.001
	Male	166 (66.14)	85 (33.86)	1	-	1	-
**Age (year)**	Less than 30	125 (60.1)	83 (39.9)	1	-	1	-
	30-50	116 (60.42)	76 (39.58)	0.99 (0.66-1.47)	0.948	0.88 (0.41-1.89)	0.739
	More than 50	59 (57.28)	44 (42.72)	1.12 (0.7-1.81)	0.635	1.14 (0.49-2.66)	0.756
**Education level**	High school diploma and lower	32 (59.26)	22 (40.74)	1	-	1	-
	Academic education	268 (59.69)	181 (40.31)	1.02 (0.57-1.81)	0.952	0.5 (0.22-1.11)	0.089
**Occupation**	Physician	47 (70.15)	20 (29.85)	1	-	1	-
	Nurse	112 (60.22)	74 (39.78)	1.55 (0.85-2.83)	0.151	0.79 (0.33-1.91)	0.6
	Non-clinical staff	141 (56.4)	109 (43.6)	1.82 (1.02-3.24)	0.044	0.65 (0.2-2.06)	0.458
**Marital status**	Single	116 (57.14)	87 (42.86)	1	-	1	-
	Married	184 (61.33)	116 (38.67)	1.19 (0.83-1.71)	0.347	1.34 (0.73-2.49)	0.348
**Living with children**	No	183 (59.61)	124 (40.39)	1		1	-
	Yes	117 (59.69)	79 (40.31)	1 (0.7-1.45)	0.985	0.93 (0.52-1.66)	0.809
**Death from COVID-19 in the relatives **	No	294 (60.49)	192 (39.51)	1	-	1	-
	Yes	6 (35.29)	11 (64.71)	2.81 (1.02-7.72)	0.045	1.89 (0.47-7.65)	0.371
**Death from COVID-19 in the colleagues **	No	266 (60.32)	175 (39.68)	1	-	1	-
	Yes	34 (54.84)	28 (45.16)	1.25 (0.73-2.14)	0.411	1.05 (0.52-2.11)	0.898
**Night shift**	No	19 (54.29)	16 (45.71)	1	-	1	-
	Yes	213 (61.38)	134 (38.62)	0.75 (0.37-1.5)	0.414	0.89 (0.38-2.06)	0.783
**History of psychiatric disorders **	No	236 (66.67)	118 (33.33)	1	-	1	-
	Yes	64 (42.95)	85 (57.05)	2.66 (1.79-3.93)	<0.001	3.6 (2.1-6.18)	<0.001
**Workplace Hospital**	Namazi	88 (61.97)	54 (38.03)	1.62 (0.8-3.27)	0.177	1.7 (0.66-4.39)	0.275
	Faghihi	83 (59.29)	57 (40.71)	1.82 (0.9-3.66)	0.096	1.26 (0.48-3.33)	0.639
	Ali-Asghar	59 (49.17)	61 (50.83)	2.73 (1.34-5.57)	0.006	1.08 (0.36-3.21)	0.895
	Dastgheib	33 (66)	17 (34)	1.36 (0.58-3.18)	0.476	1.55 (0.53-4.52)	0.42
	Hafez	37 (72.55)	14 (27.45)	1	-	1	-
**Workplace ward**	Constantly working in COVID-19 wards	57 (48.72)	60 (51.28)	4.07 (2.08-7.98)	<0.001	6.14 (2.23-16.89)	<0.001
	Episodically working in COVID-19 wards	64 (65.98)	33 (34.02)	1.99 (0.98-4.04)	0.055	2.31 (1-5.34)	0.049
	Working in non-clinical sectors	121 (56.02)	95 (43.98)	3.04 (1.62-5.69)	0.001	4.96 (1.69-14.58)	0.004
	Working in non-COVID-19 wards	58 (79.45)	15 (20.55)	1	-	1	-

**CI:** Confidence interval; **OR:**Odds ratio

**Table:** Table **2.** Association Between Socio-Demographic Features, Death from COVID-19, Psychiatric Disorders, and Workplace Features with Depression among HCWs.

**Variables**		**Depression**		**Unadjusted OR (95% CI)**	**P-value**	**Adjusted OR (95% CI)**	**P-value**	
No or mild n(%)		Moderate to High n(%)					
**Gender**	Female	163 (64.68)	89 (35.32)	0.81 (0.57-1.16)	0.26	1.15 (0.67-1.96)	0.617	
	Male	150 (59.76)	101 (40.24)	1	-	1	-	
**Age, y**	Less than 30	144 (69.23)	64 (30.77)	1	-	1	-	
	30-50	116 (60.42)	76 (39.58)	1.47 (0.98-2.23)	0.07	0.82 (0.39-1.72)	0.594	
	More than 50	53 (51.46)	50 (48.54)	2.12 (1.31-3.45)	<0.001	0.75 (0.32-1.71)	0.489	
**Education level**	High school diploma and lower	28 (51.85)	26 (48.15)	1	-	1	-	
	Academic education	285 (63.47)	164 (36.53)	0.62 (0.35-1.09)	0.1	0.45 (0.2-0.98)	0.046	
**Occupation**	Physician	47 (70.15)	20 (29.85)	1	-	1	-	
	Nurse	126 (67.74)	60 (32.26)	1.12 (0.61-2.05)	0.72	0.53 (0.21-1.29)	0.159	
	Non-clinical staff	140 (56)	110 (44)	1.85 (1.03-3.3)	0.04	0.43 (0.13-1.35)	0.148	
**Marital status**	Single	134 (66.01)	69 (33.99)	1	-	1	-	
	Married	179 (59.67)	121 (40.33)	1.31 (0.91-1.9)	0.15	1.02 (0.55-1.89)	0.96	
**Living with children**	No	203 (66.12)	104 (33.88)	1	-	1	-	
	Yes	110 (56.12)	86 (43.88)	1.53 (1.06-2.21)	0.02	1.47 (0.84-2.6)	0.179	
**Living with the elderly**	No	241 (63.09)	141 (36.91)	1	-	1	-	
	Yes	72 (59.5)	49 (40.5)	1.16 (0.77-1.77)	0.48	1.07 (0.58-1.97)	0.836	
**Death from COVID-19 in the relatives **	No	305 (62.76)	181 (37.24)	1	-	1	-
	Yes	8 (47.06)	9 (52.94)	1.9 (0.72-5)	0.2	0.92 (0.23-3.65)	0.908
**Death from COVID-19 in the colleagues **	No	281 (63.72)	160 (36.28)	1	-	1	-
	Yes	32 (51.61)	30 (48.39)	1.65 (0.97-2.81)	0.07	1.88 (0.97-3.63)	0.06
**Night shift**	No	23 (65.71)	12 (34.29)	1	-	1	-
	Yes	226 (65.13)	121 (34.87)	1.03 (0.49-2.13)	0.95	1.27 (0.55-2.94)	0.569
**History of psychiatric disorders **	No	234 (66.1)	120 (33.9)	1	-	1	-
	Yes	79 (53.02)	70 (46.98)	1.73 (1.17-2.55)	0.01	2.16 (1.28-3.66)	0.004
**Workplace hospital**	Namazi	89 (62.68)	53 (37.32)	1.94 (0.93-4.02)	0.08	1.24 (0.47-3.27)	0.668
	Faghihi	89 (63.57)	51 (36.43)	1.86 (0.9-3.88)	0.1	1.07 (0.4-2.86)	0.895
	Ali-Asghar	61 (50.83)	59 (49.17)	3.14 (1.5-6.58)	<0.001	2.11 (0.71-6.25)	0.177
	Dastgheib	35 (70)	15 (30)	1.39 (0.57-3.38)	0.460	1.29 (0.43-3.83)	0.653
	Hafez	39 (76.47)	12 (23.53)	1	-	1	-
**Workplace ward**	Constantly working in COVID-19 wards	63 (53.85)	54 (46.15)	3.96 (1.96-7.98)	<0.001	2.9 (1.05-8)	0.04
	Episodically working in COVID-19 wards	69 (71.13)	28 (28.87)	1.87 (0.89-3.94)	0.1	1.64 (0.7-3.82)	0.254
	Working in non-clinical sectors	121 (56.02)	95 (43.98)	3.62 (1.88-6.99)	<0.001	3.25 (1.13-9.36)	0.029
	Working in non-COVID-19 wards	60 (82.19)	13 (17.81)	1	-	1	-

**CI:** Confidence interval; **OR:**Odds ratio

**Table:** Table **3.** Association Between Socio-Demographic Features, Death from COVID-19, Psychiatric Disorders, and Workplace Features with Insomnia among HCWs.

**Variables**		**Insomnia**		**Unadjusted OR (95% CI)**	**P-value**	**Adjusted OR (95% CI)**	**P-value**
No n(%)		Yes n(%)					
**Gender**	Female	197 (78.17)	55 (21.83)	0.75 (0.5-1.13)	0.17	1.11 (0.62-1.99)	0.73
	Male	183 (72.91)	68 (27.09)	1	-	1	-
**Age, y**	Less than 30	157 (75.48)	51 (24.52)	1	-	1	-
	30-50	149 (77.6)	43 (22.4)	0.89 (0.56-1.41)	0.617	0.84 (0.37-1.94)	0.685
	More than 50	74 (71.84)	29 (28.16)	1.21 (0.71-2.06)	0.49	1.1 (0.44-2.74)	0.84
**Education level**	High school diploma and lower	36 (66.67)	18 (33.33)	1	-	1	-
	Academic education	344 (76.61)	105 (23.39)	0.61 (0.33-1.12)	0.111	0.42 (0.18-0.98)	0.045
**Occupation**	Physician	52 (77.61)	15 (22.39)	1	-	1	-
	Nurse	144 (77.42)	42 (22.58)	1.01 (0.52-1.98)	0.974	1.09 (0.41-2.9)	0.861
	Non-clinical staff	184 (73.6)	66 (26.4)	1.24 (0.66-2.36)	0.504	0.95 (0.27-3.36)	0.938
**Marital status**	Single	150 (73.89)	53 (26.11)	1	-	1	-
	Married	230 (76.67)	70 (23.33)	0.86 (0.57-1.3)	0.478	0.66 (0.33-1.31)	0.231
**Living with children**	No	235 (76.55)	72 (23.45)	1	-	1	-
	Yes	145 (73.98)	51 (26.02)	1.15 (0.76-1.74)	0.514	1.55 (0.81-2.94)	0.186
**Living with the elderly**	No	292 (76.44)	90 (23.56)	1	-	1	-
	Yes	88 (72.73)	33 (27.27)	1.22 (0.76-1.94)	0.408	1.26 (0.66-2.4)	0.487
**Death from COVID-19 in the relatives **	No	371 (76.34)	115 (23.66)	1	-	1	-
	Yes	9 (52.94)	8 (47.06)	2.87 (1.08-7.6)	0.034	1.78 (0.42-7.62)	0.435
**Death from COVID-19 in the colleagues **	No	336 (76.19)	105 (23.81)	1	-	1	-
	Yes	44 (70.97)	18 (29.03)	1.31 (0.73-2.36)	0.371	1.2 (0.58-2.5)	0.619
**Night shift**	No	25 (71.43)	10 (28.57)	1	-	1	-
	Yes	258 (74.35)	89 (25.65)	0.86 (0.4-1.87)	0.707	1.15 (0.46-2.86)	0.77
**History of psychiatric disorders **	No	284 (80.23)	70 (19.77)	1	-	1	-
	Yes	96 (64.43)	53 (35.57)	2.24 (1.46-3.43)	<0.001	3.67 (2.09-6.44)	<0.001
	**Workplace hospital**	Namazi	113 (79.58)	29 (20.42)	2.36 (0.86-6.48)	0.095	4.03 (1.02-15.97)	0.047
	Faghihi	98 (70)	42 (30)	3.94 (1.46-10.62)	0.007	6.18 (1.56-24.44)	0.009
	Ali-Asghar	87 (72.5)	33 (27.5)	3.49 (1.28-9.55)	0.015	4.63 (1.06-20.25)	0.042
	Dasgheib	36 (72)	14 (28)	3.58 (1.18-10.86)	0.024	7.48 (1.76-31.85)	0.006
	Hafez	46 (90.2)	5 (9.8)	1	-	1	-
**Workplace ward**	Constantly working in COVID-19 wards	81 (69.23)	36 (30.77)	2.8 (1.29-6.07)	0.009	1.78 (0.59-5.44)	0.308
	Episodically working in COVID-19 wards	75 (77.32)	22 (22.68)	1.85 (0.82-4.19)	0.142	1.32 (0.52-3.35)	0.561
	Working in non-clinical sectors	161 (74.54)	55 (25.46)	2.15 (1.03-4.48)	0.041	2.14 (0.66-6.89)	0.205
	Working in non-COVID-19 wards	63 (86.3)	10 (13.7)	1	-	1	-

**CI:** Confidence interval; **OR:**Odds ratio

**Table:** Table **4.** Association Between Socio-Demographic Features, Death from COVID-19, Psychiatric Disorders, and Workplace Features with PTSD among HCWs.

**Variables**		**PTSD**			**Unadjusted OR (95% CI)**	**P-value**	**Adjusted OR (95% CI)**	**P-value**
	Low n(%)	Moderate n(%)	High n(%)					
**Gender**	Female	62 (24.6)	130 (51.59)	60 (23.81)	1.43 (0.77-2.68)	0.257	3.8 (1.7-8.47)	0.001
	Male	84 (33.47)	119 (47.41)	48 (19.12)	1	-	1	-
**Age, y**	Less than 30	57 (27.4)	96 (46.15)	55 (26.44)	1	-	1	-
	30-50	57 (29.69)	96 (50)	39 (20.31)	2.18 (0.83-5.76)	0.116	0.86 (0.21-3.42)	0.827
	More than 50	32 (31.07)	57 (55.34)	14 (13.59)	0.79 (0.39-1.62)	0.52	0.61 (0.24-1.52)	0.288
**Education level**	High school diploma and lower	17 (31.48)	27 (50)	10 (18.52)	1	-	1	-
	Academic education	129 (28.73)	222 (49.44)	98 (21.83)	0.91 (0.36-2.31)	0.841	0.65 (0.19-2.29)	0.504
**Occupation**	Physician	22 (32.84)	24 (35.82)	21 (31.34)	1	-	1	-
	Nurse	51 (27.42)	95 (51.08)	40 (21.51)	0.69 (0.19-2.58)	0.585	0.95 (0.18-4.98)	0.952
	Non-clinical staff	73 (29.2)	130 (52)	47 (18.8)	1.7 (0.65-4.42)	0.277	0.59 (0.18-1.94)	0.383
**Marital status**	Single	49 (24.14)	100 (49.26)	54 (26.6)	1	-	1	-
	Married	97 (32.33)	149 (49.67)	54 (18)	0.77 (0.39-0.35)	0.446	0.27 (1.59-1.31)	0.656
**Living with children**	No	96 (31.27)	143 (46.58)	68 (22.15)	1	-	1	-
	Yes	50 (25.51)	106 (54.08)	40 (20.41)	2.27 (1.18-4.35)	0.014	2.92 (1.23-6.96)	0.015
**Living with the elderly**	No	119 (31.15)	181 (47.38)	82 (21.47)	1	-	1	-
	Yes	27 (22.31)	68 (56.2)	26 (21.49)	2.09 (1-4.36)	0.049	1.98 (0.78-5.03)	0.152
**Death from COVID-19 in the relatives**	No	144 (29.63)	242 (49.79)	100 (20.58)	1	-	1	-
	Yes	2 (11.76)	7 (41.18)	8 (47.06)	0.9 (0.14-5.92)	0.91	3.22 (0.42-24.48)	0.258
**Death from COVID-19 in the colleagues**	No	130 (29.48)	222 (50.34)	89 (20.18)	1	-	1	-
	Yes	16 (25.81)	27 (43.55)	19 (30.65)	1.14 (0.48-2.72)	0.77	2.16 (0.8-5.86)	0.13
**Night shift**	No	12 (34.29)	20 (57.14)	3 (8.57)	1	-	1	-
	Yes	101 (29.11)	162 (46.69)	84 (24.21)	1.24 (0.5-3.06)	0.638	5.45 (1.15-25.81)	0.033
**History of psychiatric disorders**	No	132 (37.29)	173 (48.87)	49 (13.84)	1	-	1	-
	Yes	14 (9.4)	76 (51.01)	59 (39.6)	3.13 (1.49-6.56)	0.003	10.62 (4.6-24.49)	<0.001
**Workplace hospital**	Namazi	39 (27.46)	71 (50)	32 (22.54)	1.33 (0.51-3.44)	0.557	16.72 (2.74-101.9)	0.002
	Faghihi	42 (30)	62 (44.29)	36 (25.71)	1.16 (0.44-3.06)	0.767	13.01 (2.05-82.72)	0.007
	Ali-Asghar	26 (21.67)	61 (50.83)	33 (27.5)	1.26 (0.4-3.95)	0.69	9.13 (1.18-70.56)	0.034
	Dastgheib	18 (36)	27 (54)	5 (10)	1.52 (0.54-4.32)	0.43	2.93 (0.35-24.62)	0.323
	Hafez	21 (41.18)	28 (54.9)	2 (3.92)	1	-	1	-
**Workplace ward**	Constantly working in COVID-19 wards	26 (22.22)	52 (44.44)	39 (33.33)	3.33 (1.13-9.84)	0.03	2.73 (0.68-10.91)	0.155
	Episodically Working in COVID-19 wards	23 (23.71)	50 (51.55)	24 (24.74)	4.08 (1.72-9.7)	0.001	1.51 (0.52-4.44)	0.45
	Working in non-clinical sectors	62 (28.7)	121 (56.02)	33 (15.28)	4.68 (1.5-14.58)	0.008	0.94 (0.19-4.57)	0.937
	Working in non-COVID-19 wards	35 (47.95)	26 (35.62)	12 (16.44)	1	-	1	-

**PTSD:** Post-traumatic stress disorder; **CI:** Confidence interval; **OR:**Odds ratio

## Discussion

The current study showed that mental health symptoms had a relatively high prevalence among HCWs of Shiraz hospitals during the sixth COVID-19 wave in Iran. Moderate to high levels of anxiety, depression, and insomnia among the participants. Noorbala et al. (2015) showed that the prevalence of anxiety and depression in the general Iranian population were 29.5% and 10.39%, respectively [20]. Although previous studies revealed that the outbreak of COVID-19 has also destabilized the general populations mental health, HCWs are at higher risk of presenting mental disorders [19][20].

Regarding previous studies, Iranian HCWs have expressed their prime sources of concern during the current pandemic as follows: fear of being infected by SARS-CoV-2 or transmitting the disease to their relatives, moral injury caused by being forced to share limited available resources among critically ill patients, lack of protective equipment, and failure to make the necessary arrangements and preparations to face the pandemic by the officials, which can be due to the current economic problems of Iran [21][22][23][24][25].

Regarding Hassannia et al. [25] study, anxiety and depression among HCWs were reported at 68.53% and 51.72%, respectively. Also, Azizi et al. [26] showed that 43% and 44.8% of Iranian HCWs during the COVID-19 pandemic presented anxiety and depression symptoms, respectively. However, in the current study, the prevalence of anxiety and depression were lower in HCWs.

Accordingly, it seems that over time, from the onset of the pandemic, mental health symptoms could diminish among HCWs. Studies in different countries have reported relatively different prevalence rates in assessing insomnia. While in our study, insomnia prevalence was 24.5%, a systematic review reported the prevalence of insomnia among Chinese HCWs at 38.9% [9].

However, another study in Italy estimated insomnia prevalence among HCWs as only 8.27% [8]. This relatively notable variance can be due to the differences in work shifts, workloads, and rest facilities provided to HCWs in hospitals in different countries. Also, our study estimated the prevalence of moderate to high PTSD levels at 71%, which is similar to the 71.5% prevalence reported in the study conducted by Lai et al. [9].

However, Rossi et al. [8] and Lasalvia et al. [27] estimated a relatively lower prevalence of PTSD among HCWs (49.38% and 53.8%, respectively). These findings could be due to cultural differences and the level of psychological and social support in different countries.

In our study, based on multiple logistic regression, female gender, positive history of psychiatric disorders, and constant or episodic working in COVID-19 wards over the past 12 months were associated with higher anxiety levels, which are consistent with the results of previous studies [8][9][25][28]. Fear of transmitting the disease to relatives could explain the association of living with the elderly, particularly vulnerable to the disease, with more anxiety symptoms [21][27]. A fascinating point observed in the current study was the significantly higher level of anxiety in non-clinical sectors staff. This group of employees had even higher anxiety levels than the HCWs who worked episodically in COVID-19 wards.

In contrast to our study, Lasalvia et al. indicated that the administrative staff had significantly lower anxiety symptoms [27]. The high prevalence of anxiety among non-clinical staff in our study may be due to their less scientific knowledge about COVID-19 and its transmission routes. Also, in contrast with some other studies that reported younger age as an associated factor with higher anxiety levels, our study found no such association [8][25].

Regarding our findings, some factors associated with more depressive symptoms included a positive history of psychiatric disorders, working continuously in COVID-19 wards, and working in non-clinical sectors. The association of the depression level with a positive history of psychiatric disorder and working as a front-line HCW was consistent with previous studies [9][25][26].

Also, working in non-clinical sectors of hospitals was associated with more symptoms. On the other hand, a higher education level was associated with lower levels of depression. Cohen et al., using cohort data from the National Longitudinal Survey of Youth from 1979 in the United States, showed the significant effect of higher education levels on reducing the incidence of depression [29]. Unlike other studies, which mainly reported that the female gender, being single, and younger age were associated with more depressive symptoms, no such correlations were observed [2][8][9][25][26].

Multiple logistic regression showed that HCWs with a history of mental disorders were more likely to suffer from insomnia. However, a higher education level was associated with a lower incidence of insomnia.

Previous studies confirmed that sleep disorders are associated with educational level and past psychiatric disorders [29][30][31]. However, HCWs who worked at Ali-Asghar, Namazi, Faghihi, and Dastgheib hospitals showed significantly more insomnia.

According to previous research, staff at Ali-Asghar hospital (a front-line COVID-19 hospital) and Namazi and Faghihi hospitals (second-line COVID-19 hospitals) have a higher incidence of insomnia [9]. Also, our findings revealed that the staff of Dastgheib hospital and both front- and second-line hospitals significantly had more insomnia symptoms than Hafez hospital’s staff. Although Dastgheib hospital has no COVID-19 ward, it is the largest referral center for patients with thalassemia in southern Iran. In comparison, it has a relatively higher workload than Hafez hospital.

Hence, the higher workload, in addition to direct exposure of HCWs to COVID-19 patients, could effect on insomnia prevalence.

Based on our results, female gender, living with vulnerable groups (children or the elderly), working at night shifts, positive history of psychiatric disorders, constantly or episodically working in COVID-19 wards, working in non-clinical sectors, and working in the front-line (Ali-Asghar) and second-line (Namazi and Faghihi) hospitals were associated with higher levels of PTSD.

In line with previous studies, female gender, positive history of psychiatric disorders, and working in COVID-19 front- and second-line hospitals were associated with PTSD [2][8][9][25][26][32]. In addition, since one of the most important concerns of HCWs is transmitting the disease to their relatives, the association between living with vulnerable groups and higher levels of PTSD seems reasonable [22][33]. Previous studies have also confirmed the role of history of psychiatric disorders as a predictive factor for the risk of PTSD [34]. Also, in contrast with Azizi et al. study, working in non-clinical sectors was associated with higher PTSD levels [26].

While Di Tella et al. [2] found that older age can significantly increase the risk of PTSD, Rossi et al. [8] and Azizi et al. [26] reported younger age as an associated factor with higher PTSD levels. However, in the current study, there was no correlation between PTSD and age.

Our findings demonstrated that both HCWs who worked permanently and those who worked episodically in the COVID-19 wards had significantly more mental health symptoms than the non-COVID-19 wards staff. However, the HCWs who worked episodically in the COVID-19 wards suffered from fewer psychiatric disorders.

Regarding current study results, most mental health disorders could have been associated with a prior history of psychiatric disorders, a high workload, and working on the front-line and/or even in non-clinical sectors.

Therefore, by performing periodic mental health assessments for HCWs and identifying at-risk groups, the necessary supportive and preventive measures can be taken at the right time.

Regarding Rouhbakhsh et al. study, providing adequate protective equipment, appreciation for the HCWs services, welfare facilities for the staff to stay and rest in the hospitals, training programs to deal with COVID-19 patients, and psychologist counseling for the personnel were reported by HCWs as the important factors .

Limitations

As one of the most important limitations of our study, we could not access participants mental health status before and at the beginning of the COVID-19 pandemic. In addition, despite the advantages of face-to-face interviews, there was a limited possibility of selecting a larger sample size due to being time-consuming and costly.

## Conclusion

Our study showed that mental disorders among HCWs in Shiraz hospitals was relatively high. Therefore, by conducting periodic mental health assessments, at-risk groups can be identified to be prioritized to receive supportive measures. It is suggested that specific supporting structures should be placed in healthcare systems to prevent psychological complications among HCWs.

Indeed, providing psychological and social support, and appropriate welfare facilities for the HCWs are suggested to ensure that the quality of health care services does not decline.

## Acknowledgments

The authors were grateful to employees of hospitals affiliated with the Shiraz University of Medical Sciences who participated in this study. Also, we thank of the Research Deputy of Shiraz University of Medical Sciences for financial support of the current study (grant number: 25145)

## Conflict of Interest

The authors have declared no conflict of

interest. Also, the funding body of the study did not play any role in its design, collection, analysis, data interpretation, and writing the manuscript.
